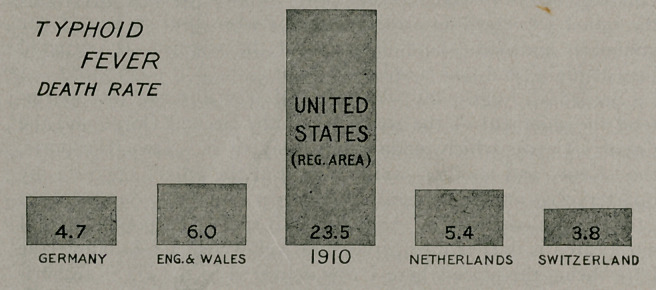# Topics of Public Interest

**Published:** 1912-10

**Authors:** 


### TOPICS OF PUBLIC INTEREST.

Typhoid Death Rates.—The accompanying illustration shows the comparative death rates per 100,000 population for various countries. It is kindly furnished by the *Human Factor*, the organ of the Equitable Life Assurance Society.

DRINKING AND SMOKING IN CANADA.—Through the courtesy of Mr. Wm. Himsworth, Acting Deputy Minister of the Department of Inland Revenue, Ottawa, we are enabled to publish the figures showing the per capita consumption in Canada of spirits, beer, wine and tobacco, for the fiscal year ended 31st March, 1912. The estimated population of Canada for that year, founded on the census of 1911, was 7,423,000.

The quantities consumed were as follows:

Spirits—7,646,834 gals.—1.030 per cap.
Beer—48,978,394 gals.—6.598 per cap.
Wine—850,017 gals.—.114 per cap.
Tobacco—27,309,604 lbs.—3.679 per cap.

For the fiscal year ending 31st March, 1911, the quantities were:—

Spirits—6,786,069 gals.
Beer—42,938,603 gals.
Wine—819,687 gals.
Tobacco—23,790,120 lbs.

The population of Canada June, 1911, according to the census, was 7,204,838.—*Canadian Journal of Medicine and Surgery.*

---

LACK OF FRESH AIR.—Dr. Livingston Farrand of the National Assn. for the Study and Prevention of Tuberculosis claims that not one person in a hundred gets enough fresh air. We are willing to concede that, at times, as at the theatre, in a street car in winter, and in various emergencies, every one suffers from inadequate ventilation and it is possible that, for a city like New York, with a dense population often affected both by poverty and ignorance, only one per cent. may have sufficient air but we are inclined to the optimistic opinion that, at present, the great majority of fairly intelligent persons enjoy plenty of fresh air at most times. However, the Association's hand-book on "Fresh Air and How to Use It," by Dr. Thomas Spees Carrington, will fill a need.

---

LECTURES AT N. Y. POST-GRADUATE MEDICAL SCHOOL AND HOSPITAL.—Prof. H. Strauss of Berlin, will lecture on Disease of the Stomach and Kidney, Oct. 12, 14 and 15th. Prof. Carl von Noorden of Frankfort, will lecture on the Pathology and Treatment of Diabetes, Radium Therapy and Arterio-sclerosis, Oct. 28th-31st.

---

HOSPITAL FOR APPENDICITIS.—The only hospital in the world thus limited, will be opened in Boston this month by Dr. Wm. A. Brooks, Jr. Although private, charity cases will be taken.

MALTA FEVER is said to prevail in Edwards and Val Verde Counties, Texas, and to have been traced to goats.

---

EQUINE CEREBRO-SPINAL MENINGITIS.—Dr. A. Boostrom, Nebraska State Veterinarian, reports that hundreds of horses have died of an epidemic—or epizootic—of cerebro-spinal meningitis in the vicinity of Holdredge.

---

GENERAL SUMMARY OF STATISTICS OF PROFESSIONAL SCHOOLS, 1910-11.

| Class. | Schools. | Instructors. | Students. | Increase (+) or decrease (—) | Graduated in 1911. | Students having a degree. |
|---|---|---|---|---|---|---|
| Theology .... | 193 | 1,495 | 10,834 | — 178 | 1,877 | 3,266 |
| Law ........ | 116 | 1,570 | 19,615 | + 48 | 3,901 | 4,180 |
| Medicine .... | 122 | 7,598 | 19,146 | —2,248 | 4,028 | 2,044 |
| Dentistry .... | 55 | 1,574 | 6,961 | + 522 | 1,764 | 122 |
| Pharmacy .... | 77 | 847 | 6,131 | — 95 | 1,743 | 84 |
| Veterinary m. | 21 | 408 | 2,571 | — 146 | 706 | 18 |

Statistics are dry on the surface, sometimes very juicy at the core. If we note that for an almost exactly equal number of students, its takes five times as many teachers in the average school of medicine as of law; or looked at another way, that the average medical faculty has about 2½ students apiece, we can understand a good many ethical and economic problems. The gist of the whole problem of medical education is that it is neither philanthropy nor business; that, for the country at large, it is obviously a matter of amateur cooperation for the sake of indirect gain or glory. Such work cannot be satisfactory from any standpoint. Let us hasten the day when the medical teacher will be paid adequately so that he can devote his best energies to teaching as a life work instead of a side-line.

---

LIGHTNING.—The Weymouth, Mass., Hospital was struck four times during a storm early in July and again on Aug. 14. Considerable damage was done by fire but no lives were lost.

The thunder storm that passed over western Pennsylvania and New York on Sept. 6, stunned a man near Jamestown, deafened a telegraph operator at Bradford and killed twin babies in Silver Creek. Curiously enough, no trace of damage was left in the room but the house next door was damaged though no one in that house was injured.

It is a fact that deserves elucidation that death from lightning occurred to one person each year in a million living in cities of the registration area for the decade 1900-09 while, for the country, the figures varied from 6 to 10 and averaged 8 per million population. (Statistics courteously furnished by E. Dana Durand, Director of the Census). Last summer, between Detroit and Ann Arbor, we noticed quite a number of lightning rods which have gone out of fashion in Western N. Y. It occurs to us that the whole matter of danger from lightning, to human beings, domestic animals and property, is well worth thorough scientific investigation.

---

ANTERIOR POLIOMYELITIS.—Cases of this disease are being reported in Buffalo and many surrounding towns. On Sept. 6, Dr. Fronczak called a mass meeting of physicians in the Common Council Chamber. More than 400 persons, mostly physicians, responded. The following program was presented:

Dr. Wade H. Frost, Past Assistant Surgeon of the United States Public Health Service—"Epidemiology and Etiology."

Dr. Frank A. Fraser, Clinician in the Hospital attached to the Rockefeller Institute for Medical Research—"Pathology, Symptomatology, and an outline of the work and the results obtained in the Institute."

Dr. Nelson G. Russell, of the Ernest Wende Hospital Staff—"Diagnosis and Treatment."

Dr. Edward A. Sharp, of the Ernest Wende Hospital Staff—"Presentation of Cases."

Dr. James P. Leak, of the United States Public Health Service, and Dr. Harlan P. Cole, Consulting Orthopedist of the New York State Department of Health, also addressed the meeting.

On consultation with several of the speakers it was decided not to attempt to reproduce the remarks, even in abstract, as the object was merely to call attention to the epidemic and to review well known facts regarding the disease. The Supt. of Education, Henry P. Emerson, called attention to the fact that school registration had diminished through fear of the disease and he asked Dr. Fronczak as to the actual danger of contagion. The reply of the latter was to the effect that the general opinion of the profession held the danger of contagion as slight.

We recommend the general tendency of the press, the profession and the various "experts" sent to Buffalo to study the epidemic, to regard matters in a conservative way. Anterior Poliomyelitis is by no means a new disease nor has there been any great increase in its prevalence of late years. While a student at the University of Pennsylvania in 1888-9, we received almost exactly the same teaching as to its nature as some consider distinctly modern. That is to say, its pathology was well understood and it was regarded as probably of bacterial origin, possibly even due to a specific germ, but not yet demonstrated. Regarding the supposition of an "ultra-microscopic" germ, we would say that most undetermined diseases have been ascribed to such causes, that several apparent demonstrations of ultra-microscopic virus have been shown to be due to leaky porcelain filters and that the treponema of syphilis is a good deal larger than most specific disease germs, although this disease was once stated to have been demonstrated to have an ultra-microscopic cause.

Dr. Fraser pointed out that the disease attacked the anterior horns largely on account of the anatomy of the blood vessels and that much of the initial paralysis was due to hyperaemia and active œdema. It is just possible that there is such a process as non-bacterial inflammation and that this disease will prove to be of such nature. However, we are inclined to the infective theory though not necessarily to the idea of a specific germ.

---

Our foreign exchanges state that a movement is on foot in France to augment physicians' earnings, on account of rise in cost of living. One estimate is that an unmarried French physician must earn $1700 a year to pay expenses. This statement we doubt very much. In 1908, one or our medical friends in Paris, a bachelor of about 60 years, an expert in cardiac diseases, regarded as successful for one not a celebrity, spoke of his daily average earnings of 50 francs, with frank pride. He occupied an apartment near the Place of the Star, at about $60 a month, fairly comparable in desirability and price with similar apartments in large American cities. Speaking generally, the cost of living in Paris is about two-thirds what it would be in Buffalo, Rochester, Syracuse, etc. Not only can an unmarried physician live in the United States for less than $1700, but the average of all physicians, married or single, do live on considerably less, according to all estimates that we have seen. Whether there is the same proportionate difference in cost of living between large and small towns in France as in America, we cannot say, but we are very sure that an unmarried physician can live on $1200 a year in America in a fair degree of comfort and that he could live equally well in Paris on $800. But we are not pleading that he ought to be compelled to do so.

---

Anti-Typhoid Vaccination.—Dr. W. H. Watters, Director of the Dept. of Pathology and Bacteriology, Evans Institute for Clinical Research, Boston, is collecting data. He requests references to published articles and unpublished case reports for which special blanks will be furnished.

Prize Essay on Therapeutics.—The American Therapeutic Society offers prizes of $250, $150 and $100 for the best three papers on some substance official in the U. S. P., submitted before April 1, 1913. The usual conditions as to secrecy, originality, standard of merit, typewriting, etc., are in force. Address Dr. Reynold Webb Wilcox, Chairman, N. Y. City.

---

Historic Exhibition of Rare and Curious Objects Relating to Medicine, etc.—Mr. Henry S. Wellcome, the well known pharmaceutic manufacturer is organizing such an exhibit at the time of the International Medical Congress in London in 1913. His own research and collection are of the highest value and he bespeaks the assistance of physicians and others all over the world.

We take the liberty to suggest that exhibits of this nature, on a small scale, necessarily, might well be undertaken by various societies in our own territory. The editor has several medical books from one to three centuries old and many of his acquaintances have larger collections. Dr. Park's collection of copies of mediaeval pictures bearing on medicine and surgery is of great value. Old instruments, signs, record blanks, etc., even if not ancient, would be of local interest. From our whole territory, a very considerable exhibit could be gathered which might be passed from city to city for inspection and discussion. Perhaps some of our readers can augment the editor's personal collection of Indian bones showing fractures, arrow head wounds, syphilitic (?) lesions, and anomalies. We would like to see the best of such a collection of local antiquities in London, next year.

---

The Goat Industry.—Apropos of Mr. Bull's book on the Goat, reviewed in our May, 1912, issue, the report of U. S. Consul, Canada, (his name, not a misprint) at Vera Cruz, Mexico, furnishes strong corroboration of Mr. Bull's plea for goat raising in the U. S. Mr. Canada refers to the feasibility of raising goats on arid land, unsuited for other purposes and says:

The value of the goat depends upon three things, meat, tallow, and the skin. * * * The matter of fresh meat in the farming districts of the hot country is greatly simplified by the breeding of goats. In small, isolated communities it is risky to slaughter an ox or cow, as the flesh is liable to spoil before it is consumed. The small carcass of a goat makes it entirely practicable to kill frequently without risk or loss. In the State of Tobasco the inhabitants are never at a loss for fresh meat on plantations where goats are raised, and where formerly meat was almost unknown. * * * The flesh alone would make it a profitable industry, for the carcass of each animal will bring from $1 to $1.50. Fats of all kinds are high priced in all parts of the country. * * * At two years a fat goat will yield 6 to 10 pounds of clear tallow, which will be worth from 75c to $1.50. * * * The most valuable part of the goat is the skin, worth $1.25 to $1.50, and the constant trend of prices is upward." (These figures of values are presumably in Mexican currency, in which the dollar is equivalent to about one-half dollar in United States currency).

The Consul adds that the increase in the number of goats is very rapid, since they begin breeding at from 6 to 8 months of age, and breed twice a year, bringing forth two and not infrequently three kids at each breeding season. A single shepherd it is said will look after at least 1,000 goats. He adds: "A careful investment of $1,000 well looked after can certainly be counted upon to double itself inside of 2½ years, and to give at least a 50% profit per annum from the end of the second year." The report of Consul Canada closes by presenting the result of a three years experiment in goat raising on a farm in the State of Guerrero, Mexico, as follows:

"In 1904 the manager of the farm received 66,000 goats, including large and small, at a valuation of $1.50 each, equal to $99,000. From the produce of these he sold, during 9 months of 1904 and the years 1905 and 1906, 50,000 head at $5 each, equal to $250,000, and had, at the close of 1906, 88,000 head on the farm, being 22,000 more than he started with. The result was, counting the value of the 22,000 increase at $1.50 each with the foregoing sales, the investment of $99,000 produced in less than three years $283,000, besides the original stock of 66,000 goats was intact." (Figures in Mexican currency.)

The above facts as to the possibilities in the increase of the meat supply of the United States, while retaining at home the hundreds of millions of dollars now sent abroad for the purchase of goat skins, and producing this supply of valuable material on lands now practically unutilized, are cited as the cause of the numerous inquiries upon this subject now reaching the Department of Commerce and Labor. The number of goats now produced in the United States is small compared with that of many other countries. The latest figures in the Bureau of Statistics show the number in the United States in 1910 at about 3 million; in Mexico, 4¼ million; Turkey in Asia, 9 million; British South Africa, 12 million; and British India, 34 million.

Furthermore, it is probable that the availability of goat meat and milk will prove of great economic and humanitarian value in preventing tuberculosis.

Statistics of Medical Education.—In accordance with a precedent now several years old, the *Journal of the A. M. A.* in its issue of Aug. 24, 1912, presents an elaborate report on various phases of medical education, which deserves the careful study of every medical man. Last year, the "non-descript" medical schools disappeared from the list; this year, "physio-medical" have disappeared; eclectic schools have decreased to 6 and homoeopathic to 10, leaving an even hundred regular schools. Altogether, the colleges have decreased from 166 in 1904 to 116.

The graduates, who have declined almost steadily from the high mark of 5747 in 1904, showed a slight increase from 4273 in 1911 to 4483 in 1912. However, the total number of medical students has declined from 28,142 in 1904 to 19,786 in 1911 and 18,412 in 1912. Thus it seems almost certain that next year's class will drop lower than in 1911. But it is interesting to note that while, with the larger numbers of students and some colleges still giving only three year courses, less than one-fifth of the total number of students graduated, at present, very nearly a quarter graduate, as should be the case with four-year courses throughout and no waste of time by men passing from one plan of life-work to another.

Almost exactly 10% of the total graduations were from New York State colleges: 429; and this state also contains about 10% of the licensed profession: 13,641, according to the directory prepared by the state in 1911. About 2% of the profession die annually; the increase in total population and hence for the relative need of physicians is about 2% and, so far as can be judged from a careful local study, about 5% of the profession, as listed, is not in practice. Hence, it is obvious that the average clientele is increasing, but very slowly. Now is the time to restrict still further the output of unneeded physicians.

---

Vehicular Fatalities.—The National Highways Protective Society reports 20 children and 28 adults killed in New York City in August, 1912, about double the number killed in August, 1911. 18 deaths were caused by automobiles, 13 by trolleys and 17 by wagons. 263 persons were injured by vehicles. In New York State, outside of New York City, the latter having a little over half the total population, 17 deaths were due to automobiles, 12 to trolleys, none to wagons. 209 traumatisms occurred, 175 due to automobiles, 12 to trolleys, 20 to wagons.

A good many deaths directly due to street cars and horse-drawn vehicles are undoubtedly due to attempts to dodge approaching autos, of which most persons have an especial and perfectly justified fear. Previous statistics have shown that automobiles have, for nearly ten years, caused more deaths than street and railroad cars combined. The humble bicycle, so much feared a few years ago, seems to have lost its death-dealing powers, or rather it was formerly rated as much more dangerous than it really was. Children will be killed by vehicles, in spite of the utmost care by chauffeurs, motor men and drivers, just as long as they are allowed to use the streets as a play ground.

---

CENTENNARIANS.—David Hancock of Red Wing, Minn., celebrated his hundredth anniversary on Apr. 12, 1912, with his comrades of the 3d Minn. Infantry. He was past the age for enlistment during the Civil War but the lieutenant colonel of the regiment took a few years off his age. The *Boston M. & S. Jour.*, of Aug. 29, 1912, reports a man and a single woman who have recently passed the hundredth birthday, a married woman who was 106 on Aug. 26; and another nearly 102.

---

THE INTERNATIONAL JOINT COMMISSION will meet in Ottawa, Oct. 1, to begin active work on the problem of pollution of the Great Lakes, tributary and communicating streams. We have elsewhere presented statistics of typhoid incidence. Various European cities have typhoid death rates of 1 to 8 per hundred thousand population. Niagara Falls averaged 129 for the ten years 1898-1909 and ran up to 222 for 1907. We personally believe that the million and more tourists to the Falls every summer disseminate typhoid over the whole country just as truly, though not quite so spectacularly as pilgrimages to Mecca have disseminated cholera Asiatica. It is not yellow journalism but a very conservative statement and probably far below the ultimate total, to say that this commission holds in its hands the lives of a thousand human beings a year.

---

NIAGARA WATER.—Lockport and the Tonawandas are protesting against the gradual filling in of the channel west of Motor (nee Frog) Island, claiming that the shutting off of the current from the west side of the river renders the pollution by Buffalo sewage more noticeable. The contention is entirely correct and the channel should not only remain open but should be dredged to a navigable depth. However, we would advise the down-stream cities not to depend upon the displacement of the polluted eastern current by the re-establishment of the clearer inflow from the west but to filter, or boil the water or to introduce general anti-typhoid vaccination until we are civilized enough to keep our sewage out of the river.

Assistant in Experimental Therapeutics, Philippine Service. Eligibility will be determined by credentials as to attainments and experience. Initial salary $2,000. Apply for Form B.I.A. 2, of U. S. Civil Service Commission. Applications must be returned complete, by Oct. 11.

---

The new building of the German Deaconess Hospital of Buffalo, will be formally opened Oct. 1. Receptions will be held for several days before the patients will be transferred. The old building will them be used as a nurses' home and to accommodate aged persons not under active treatment.

## Figures and Tables

**Figure f1:**